# A prototype RFID tag for detecting bumblebee visitations within fragmented landscapes

**DOI:** 10.1186/s13036-019-0143-x

**Published:** 2019-02-07

**Authors:** Sarah E. Barlow, Mark A. O’Neill, Bruce M. Pavlik

**Affiliations:** 1 Royal Botanic Gardens, Kew, Surrey, TW9 3AB UK; 2 Tumbling Dice Ltd, Newcastle upon Tyne, NE3 4RT UK; 3 Present address: Red Butte Garden and Arboretum, University of Utah, Salt Lake City, UT 84108 USA

**Keywords:** *Bombus*, Radio tracking, Insect flight, Movement ecology, Pollinators, Telemetry

## Abstract

Detecting the arbitrary movements of fast-moving insects under field conditions is notoriously difficult because existing technologies are limited by issues of size, weight, range and cost. Here, we establish proof-of-concept for a prototype long-range, passive radio frequency identification (RFID) tagging system for detecting bumblebees and similar sized insects. The prototype tags, weighing 81 mg (49% of mean bee body weight), were flown by bumblebees in a glasshouse and detected at a distance of 1.5 m from a 2 W UHF reader with two aerials. This detection distance is two orders of magnitude greater than existing RFID tags that can be flown by medium-sized bees and, thus, is a significant breakthrough for insect tracking that could be applied to plant conservation and restoration efforts in fragmented landscapes.

Proof-of-concept has been successfully established and, with further development, we are likely to optimize the system by reducing tag size and weight to limit effects on bee behaviour, and by increasing the detection distance. We envisage the production system being used to detect and track bee movement pathways within a designed network of field-deployed low-cost readers and aerials. The production system could be used in a wide variety of scientific and commercial applications.

**Electronic supplementary material:**

The online version of this article (10.1186/s13036-019-0143-x) contains supplementary material, which is available to authorized users.

## Background

Studying insect movement, migration and behaviour can help provide answers to important ecological questions that impact the diversity, health and persistence of species and ecosystems [1]. Unravelling the enigmas around global pollinator declines is one such example. However, the rapid movements of flying insects, such as bees, are notoriously difficult to rigorously document. Although useful insect telemetry techniques exist they are limited by issues of transmitter size, range, reliability, and overall cost for the purposes of tracking complex activity at landscape-scales [1, 2]. Scientists have used radio frequency technology to track larger animals, such as birds and mammals [1, 2], but the battery-powered (active) radio transmitters are too big and heavy to be carried by all but the largest flying insects (e.g. UHF radio transmitters weighing 250–300 mg have been used to track large hawk moths [3] and orchid bees [4]). The trade-off being that a power supply will increase a transmitter’s range but will also increase its weight and size. Innovation in radio tracking systems is needed to reveal more about the movement ecology of small- and medium-sized insects in the real world of fragmented landscapes.

Passive radio frequency identification (RFID) tags lack a power supply and have been successfully used to monitor insect activity in some studies. For example, passive RFID tags weighing ca. 3 mg and having a sub-cm detection range have been used to detect honeybees and bumblebees leaving and returning to hives and visiting feeding stations [5, 6]. However, the severely limited detection distance of these tiny tags means that bees must pass very close to a reader (positioned at the hive or baited feeding stations). By comparison, harmonic radar has been used to track the trajectories of bumblebees and honeybees up to 900 m [7, 8] and Asian yellow-legged hornets (*Vespa velutina*) up to 150 m from a receiving antenna [9]. Although this system has proved useful there are some limitations. Those harmonic radars require a passive transponder with a 16 mm long vertical loop antenna to be attached to an insect’s back, effectively preventing the insect from accessing its nest and foraging from some flowers. Other drawbacks are that the transponder signal is not uniquely identified, and the signal is temporarily lost if the insect flies behind an intervening object [7–9]. Harmonic radar is also relatively very expensive rendering it inaccessible to most researchers.

Herein we introduce a new prototype tag that uses passive RFID technology. Our aim in this proof-of-concept study was to develop a lightweight, long-range tag suitable for detecting bumblebees at a range of 1 m (i.e. ≥100-fold increase in the detection range of existing passive tags flown by bees). We set this goal because it is a feasible range for detecting bees visiting flower patches and for mapping patch connectivity in the field (essential for genetic exchange in plant populations) with a designed array of multiple readers and aerials. As *Bombus terrestris* foragers may weigh in the region of 200 mg or more [7], and are able to carry 90% of their body weight in nectar and pollen [10], we aimed to develop a prototype tag with an upper weight and size limit of ca.135 mg and 10 mm × 15 mm respectively to test proof-of-concept, but envisaging a much lighter “production” tag in due course weighing substantially less than the average pollen and nectar load (50% of body weight [7],) and similar in weight to harmonic transponders flown on bumblebees and honeybees (ca. 12 mg [7, 8],). Furthermore, the system was designed to use COTS (commercial off-the-shelf) technology including relatively low power, low cost aerials, and have a user-friendly control interface capable of detecting and displaying information as unique, time-stamped visits. The system must be low cost because large quantities of expendable tags and multiple aerials would be required to satisfy our intended uses under field conditions and be obtainable to researchers. To establish proof-of-concept, we assembled tags by hand and tested the new system with free-flying bumblebees under glasshouse conditions. We present our findings and offer our thoughts on future directions of the technology.

## Prototype tag design and proof-of-concept testing

### Tag components and assembly

**Fig. 1:**
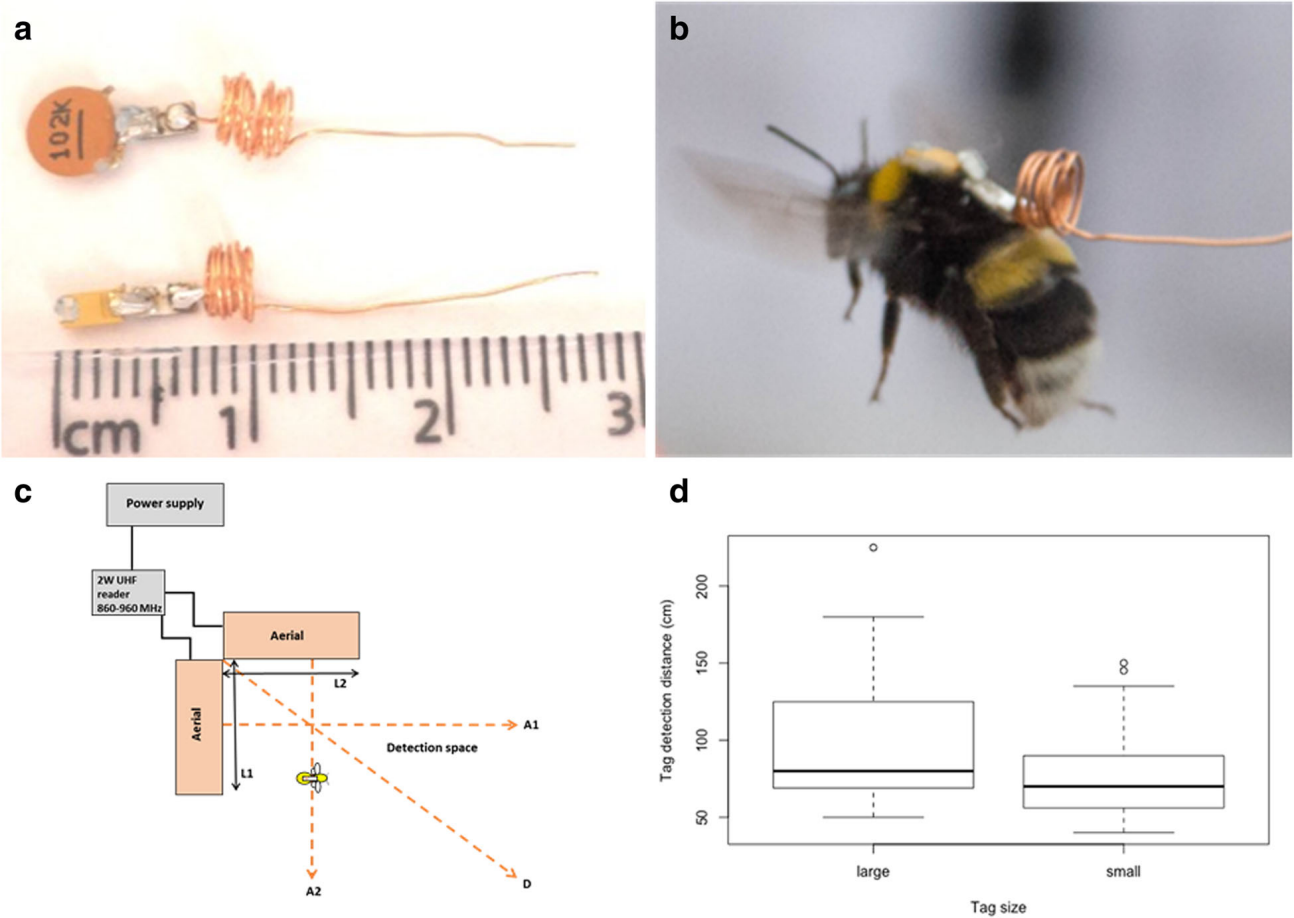
Proof-of-concept testing of prototype RFID tags attached to bumblebees in a glasshouse environment. **a** Two sizes of prototype passive RFID tag comprising a muRata MAGICSTRAP® chip and hand-coiled inductively loaded whip antenna. The smaller tag (btm) is 7 × 2 mm, plus a 20 mm coiled whip antenna, and weighs 81 mg. The larger tag (top) weighs 128 mg. **b** An in-flight *Bombus terrestris* worker carrying a prototype RFID tag. **c** Experimental design of tag testing arena and tracking system hardware. A1, A2, D = tag detection distance recorded at three approach angles from two aerials. L1, L2 = 52 cm. **d** The larger tag has a significantly greater detection distance than the smaller tag (t-test: *t* = 2.97, *P* < 0.01). Maximum detection distances of large and small tags from two orthogonal aerials were 225 cm and 150 cm, respectively. N_large_ = 12, N_small_ = 20 tested at three approach angles

The prototype tags use a COTS RFID strap chip (muRata MAGICSTRAP® LXMS31) with a custom inductively loaded, half dipole whip antenna which is well suited to RF coupling (Fig. 1a). To energise the tag and receive its transmission, we used a 2 W UHF long-range reader (receiving frequency 860–960 MHz) (ID ISC.LRU1002 reader, Feig GmBH, Weiburg, Hessen, Germany) equipped with two antennas. To avoid confusion, hereon we refer to a tag’s antenna as an “antenna” and the receiver’s antennas as “aerials”. Tags of two sizes were made by hand hence, for each tag size we expected to find marginal variation in tag weight (due to soldering) and transponder detection distance (due to variation in the hand-coiled whip antenna). Note, these issues will disappear if tags are fabricated by machine in future as intended. For the whip antenna, we hand-coiled copper wire, 0.5 mm in diameter and ca. 15 cm in length and trimmed until RF resonance was achieved. The smaller tags are 7 × 2 mm, plus a 20 mm coiled inductance, and weigh 81.2 ± 4.97 mg (mean ± SEM). Larger tags have a larger ceramic capacitor and weigh 128 ± 5.66 mg (Fig. 1a).

### Study species and test conditions

To provide representative data and validate proof of concept we tested the prototype tags on worker bumblebees (*Bombus terrestris audax*) within a controlled glasshouse environment at the Royal Botanic Gardens, Kew. Within a glasshouse, we constructed a bee tent of fine mesh netting suspended on wire cables (measuring 8 m (L) × 3 m (W) × 2.5 m (H)) to contain the bees during trials. A commercial bumblebee colony (Agralan, UK) containing artificial food supply was introduced to the bee tent, along with pots of flowering “bee-friendly” plants (*Salvia*, *Lavendula*), and bees were allowed to acclimatize for three weeks. During this time, bees were able to fly freely and visit flowers, and experienced low levels of mortality.

### Tagging bees

A subset of worker bees was captured, weighed and chilled at 5 °C for 15–20 min. Bees were then secured to a polystyrene board with pins arranged over the gaster and between tibias before applying a small amount of fast-drying epoxy resin (Araldite) to the centre of the bee’s thorax to permanently glue the tag in place (Fig. 1b).

### Proof-of-concept testing

To test the detection distances of the tags, we positioned, in the bee tent, a 2 W UHF reader, its two aerials (at ground level), a power supply and control laptop. The maximum detection distance of tags was measured at three approach angles (90 degrees at aerial midpoint and diagonal) from the aerials arranged orthogonally (L-shape) between 7 and 14 cm above ground (Fig. 1c). Two aerials arranged in an L-shape make a quasi-omnidirectional detection space. Four aerials spaced apart, or critically placed microwave reflectors, may give a larger detection space, but this was not tested here.

Tags were tested before (attached with sticky [blu] tack to a ruler) and after being glued to bees. Individual tagged bees were placed in a small open plastic box and positioned within the detection space as described above. Some tags were broken and could only be tested once as the whip antenna proved to be fragile (note, the next version of the tags will be more robust by using a solid-state inductance module and shorter antenna). Other tags were tested multiple times as minor adjustments were made by hand to the whip antenna, hence we recorded variation in maximum detection distances for the same tags (i.e. each time a tag was adjusted it was re-tested) (n_large_ = 12, n_small_ = 20 tested at three approach angles). During testing, we evaluated the usability of the control interface.

We also observed the flight and behaviours of tagged bees for several hours over three consecutive days to determine the effects of the tags, the glue and the handling. A subset of tagged bees was retained and monitored daily for a further five weeks.

## Results and discussion

### Effective tag detection (transmitter) distance

Larger tags had a greater detection distance than small tags because the dipole ballast capacitor was of higher value (t-test, *t* = 2.97, *P* < 0.01; Fig. 1d). On average (mean ± SEM), large and small tags were reliably detected at 99.7 ± 7.3 cm and 75.7 ± 3.6 cm from the aerials, and maximum detection distances were 225 cm and 150 cm, respectively. For each size of tag, variation in detection distances was due to small differences in the width and length of the whip antenna coil as tags were made and adjusted by hand. This variation can be reduced in a subsequent version of the tags by machine fabricating an optimised antenna. The approach angles of a tag in relation to an aerial did not affect the detection distance because the orthogonal aerial arrangement generates an omnidirectional detection space (Kruskal-Wallis test: approach angle, large tag, chi^2^ = 3.31, *P* = 0.191; small tag, chi^2^ = 0.91, *P* = 0.64), although the signal was marginally stronger at 90 degrees from an aerial. Note, that with a single aerial, the tag would show directional dependency. Four aerials optimally arranged (or aerials paired with microwave reflectors) might increase the maximum detection range significantly. Clutter (produced by solid objects near the detection space including people, plants, etc.) was found to influence the detection distance as well.

### Control interface

The control interface displayed the data output as a flat ASCII text file showing tag ID code, date and time effectively generating a record for every tagged bee entering the detection space. With further development, the control interface will have additional features (see Additional file 1).

### Effects on bee behaviour

Bees weighed 167.2 ± 7 mg (mean ± SEM, *n* = 10), thus, on average, small and large tags weighed 49% and 77% of bee’s body weight, respectively. Immediately after being tagged, bees were able to walk and feed on sugar solution, but only those bees fitted with small tags were able to take-off and fly normally. Initially, bees unsuccessfully attempted to remove tags with their legs. Tagged bees were left in the bee tent but did not return to the nest during the subsequent 3-day observation period. Of the bees that were tagged, five (three with large and two with small tags) were retained for further monitoring, fed on honey, and were still alive five weeks after tagging. One complication was entanglement of the tag antennas between tagged bees, which was more likely to occur when confined to a small space (and would be particularly problematic inside a nest). We recognise, therefore, that the prototype tag must be improved upon in this regard and further experiments are necessary to test the effects of tag attachment on bee behaviour.

## Future goals

We have developed an insect detection system that uses novel passive RFID tags of a weight (81 mg) and spatial footprint (7 × 2 mm) that can be ‘flown’ on *Bombus terrestris* and detected up to 150 cm from a 2 W reader. This constitutes a significant breakthrough as this detection distance is two orders of magnitude greater than existing passive RFID tags used for similar purposes (e.g. honeybees [5]; bumblebees [6]). Our larger tag (128 mg) had a longer detection distance, up to 225 cm, but was too heavy and impeded bee flight (although it might be useful for larger insects).

Small tags allowed bees to take off and fly normally but would likely impede nesting and foraging behaviours (e.g. reducing the nectar and pollen load that bees can normally carry). As such, the prototype requires improvement and we have identified several optimization goals, although due to the sensitive nature of this nascent technology, we are unable to give specific details on how these could be achieved. We expect that the next generation of our tracking system will include a tag with all components except the antenna integrated on to a single silicon die, and the inductance will be a machine-fabricated solid-state component (< 1 cm straight antenna, horizontally-mounted) as opposed to a whip antenna with hand-wound coil. The precise design will be informed by iteratively modelling the effective circuit using a physics simulation package (e.g. COMSOL) to determine the optimal values of L (inductance) and C (capacitance) for the inductively loaded antenna. Thus, a production tag would weigh much less (possibly ca. 10 mg) and allow the technology to be tested with smaller insects such as honeybees, some syrphids and solitary bees. Further, we will evaluate the potential benefit of a 4 W reader and of using an optimal array of four aerials (and/or two aerials plus microwave radiation reflectors) in increasing the radiation density within the detection space, and, hence, tag detection distance. Finally, the control interface will be able to log detection events from multiple readers, thus allowing bee movement pathways to be reconstructed through space and time. These developments could then be tested in the field including more rigorous experiments of how tag attachment effects bee flight, foraging and nesting behaviours.

**Fig. 2:**
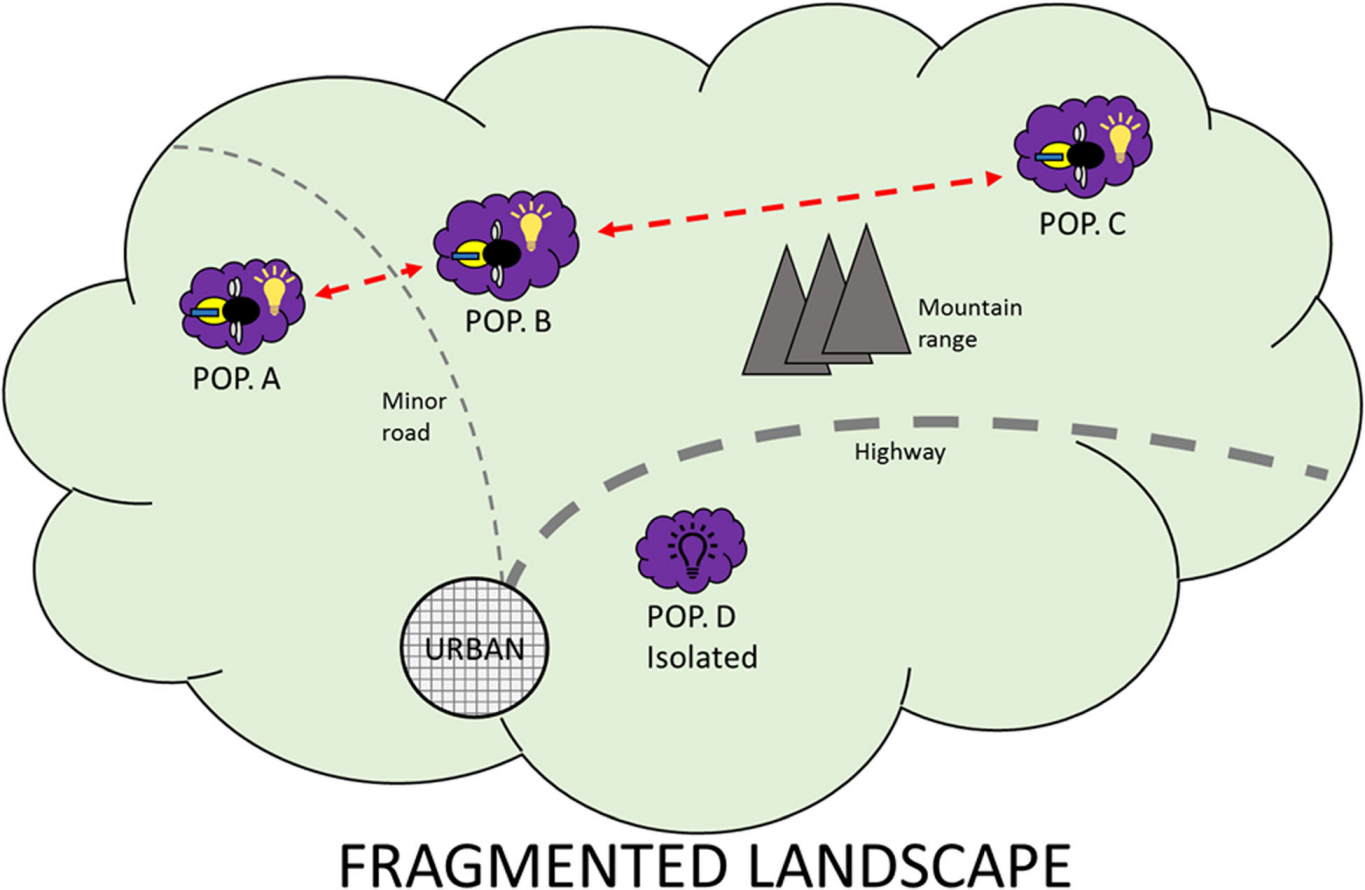
Measuring ecological connectivity using tagged bees and readers to integrate movement and landscape ecology for conservation management. The same tagged bee(s) connects three of four populations of a plant species, resulting in pollen (gene) flow among A, B and C. A confirmed lack of visitation to D (due to, for example, a wide highway) means the population will degrade over time for lack of gene flow and be of little conservation value unless restorative actions are taken. The effectiveness of those actions could then be monitored using the same tag detection system

Ultimately, tags and readers would be deployed in the field to solve problems in plant conservation and restoration. The fragmentation of natural landscapes by human activities (e.g. agriculture, road construction, deforestation) has reduced and isolated populations of once continuously distributed species. Pollinating insects that once distributed pollen and genes across a species’ range may not cross such artificial barriers, resulting in a slow degradation of isolated populations. Being able to measure visitation among populations with these tags would identify barriers to pollen movement, distinguish between healthy and ailing populations and provide a means of measuring the effectiveness of subsequent restoration efforts (Fig. 2).

## Conclusions

In conclusion, testing the prototype RFID tags on free-flying bumblebees has successfully established proof-of-concept. The prototype is the vanguard of our new detection and tracking system and further optimization seems feasible. If successful, we intend production tags to be used with a designed network of low-cost readers to build maps of bee flight paths in response to landscape factors and gain new insight into landscape-scale phenomena. As ecologists, we envisage using the technology for bee and plant conservation purposes, for example we need to better understand pollen (gene) flow between bee-pollinated plant populations, and bee range and habitat requirements at landscape-scales, but see many opportunities for using the new tags in studies of insect and plant science (for example, experiments of the impacts of pests, diseases and pesticides on bee health; the impacts and spread of alien species; and gene flow associated with crops and wild relatives). We also intend tags to be low cost and, therefore, expendable and interoperable with existing COTS (commercial off-the-shelf) technology. As such, the new technology has wide potential for scientific application and, in time, we anticipate the system to be made commercially available.

## Additional file

Additional file 1: Intended further development of the control interface. (DOCX 15 kb)
