# Supplementary material for: A prototype RFID tag for detecting bumblebee visitations within fragmented landscapes
Source: J Biol Eng. 2019 Feb 7;13:13. doi: 10.1186/s13036-019-0143-x (PMC6367791; doi:10.1186/s13036-019-0143-x)
Supplement: Supplementary file 1 — Intended further development of the control interface. (DOCX 15 kb) [file 13036_2019_143_MOESM1_ESM.docx]

**Additional file 1: Intended further development of the control interface**

*Control interface*

With further development, the fully functional control interface will allow the user to: 1) amend time and date (on/off clock set); 2) start/stop recording by the base station; 3) know the GPS co-ordinates of the base station; 4) select sleep controls (to conserve power at night); 5) select tag filtering options (e.g. to track subgroups of a tagged cohort of bees); 6) select scanning controls (to re-scan the zone at timed intervals (minimum 1 second intervals) producing data pertaining to how long the tagged individuals remain within the zone); 7) select a gain control (to control the size of the detection zone such as a floral patch monitored for pollinator activity); 8) select filter controls (to view data according to date and time of detection events or per tagged individual); and 9) remotely access and download data from the base station via a WLAN connection.
